# Trends in global shark attacks

**DOI:** 10.1371/journal.pone.0211049

**Published:** 2019-02-27

**Authors:** Stephen R. Midway, Tyler Wagner, George H. Burgess

**Affiliations:** 1 Department of Oceanography and Coastal Sciences, Louisiana State University, Baton Rouge, LA, United States of America; 2 U.S. Geological Survey, Pennsylvania Cooperative Fish and Wildlife Research Unit, Pennsylvania State University, University Park, PA, United States of America; 3 International Shark Attack File; University of Florida, Gainesville, FL, United States of America; Aristotle University of Thessaloniki, GREECE

## Abstract

Shark attacks are a global phenomenon that attracts widespread attention and publicity, often with negative outcomes for shark populations. Despite the widespread perceptions of shark attacks, trends in human water activities and shark populations are both dynamic, resulting in variable rates of shark attacks over space and time. Understanding variable trends in shark attacks may contribute to a better understanding of risk, and a more tempered response in the wake of an attack. We found that global shark attack rates are low, yet variable across global regions and over decades. Countries with low populations were found to have the highest rates of attack, while countries with high populations (U.S.A., Australia, South Africa) tended to have overall low attack rates, but also much more interannual variability. From the 1960s to the present, those countries with the highest populations also tended to be the places where attack rates have increased. Ultimately, shark attack risk is also driven by local conditions (e.g., time of day, species present); however, a global scale understanding of attack rates helps place risk into perspective and may contribute to a more scientifically-grounded discussion of sharks, and their management and conservation.

## Introduction

Sharks are a diverse group of cartilaginous fishes that have drawn considerable scientific and popular interest. Although most often associated with marine habitats, some species occupy brackish and even freshwater habitats [1, 2], and in many systems shark species are considered meso- or apex-predators [3, 4]. The equilibrium life history strategy [5] of most shark species leaves them particularly vulnerable to fishing mortality. While sharks are valuable contributors to fisheries around the world [6], their life-history limitations have contributed to resultant overfishing in most directed shark fisheries and in many multi-species fisheries that feature high bycatch of sharks [7]. Our knowledge of shark population dynamics is limited compared to that of many teleost species, although good examples do exist of shark population monitoring–e.g., Bimini lemon sharks (*Negaprion brevirostris*), Gulf of Mexico blacktip sharks (*Carcharhinus limbatus*), and the Australian gummy shark (*Mustelus antarcticus*). Because of gaps in biological and population data, broad inferences occasionally have been drawn from limited data [8, 9], leading to contrasting evaluations of populations [10] which unfortunately promotes confusion surrounding the status and trends of individual shark populations. Despite the instances of confusion, there are examples of shark populations that are and are not doing well [11].

Sharks also are of interest to humans because they represent one of only a few groups of animals that negatively interact with *Homo sapiens* on a regular, albeit uncommon, basis through the phenomenon commonly referred to as “shark attack.” Although most of these interactions result in minor injuries akin to that of a dog bite, about six of the 75–100 unprovoked attacks that currently occur worldwide each year result in human mortality [12]. (Note that an *unprovoked attack* is defined as a bite or near bite (fended off by human intervention) of a person (or the board on which he/she is perched) in the shark’s natural environment in the absence of any human provocation.) Despite its relative rarity, shark attack is a cultural phenomenon that draws intense public interest in the popular media [13] with myths and misconceptions routinely perpetuated on television, in magazines and newspapers, and in the social media.

Three shark species that attract much of that attention are the bull shark (*Carcharhinus leucas*), tiger shark (*Galeocerdo cuvier*), and white shark (*Carcharodon carcharias*). These species are of interest to biologists because they are large, migratory species of cosmopolitan distribution; however, they are also widely-known species in the public sphere owing to their depiction in movies, charismatic megafauna status, and for their association with reports of fatal shark attacks. The IUCN (International Union for Conservation of Nature) Red List has classified white sharks as Vulnerable and bull and tiger sharks as Near Threatened, although the status of individual populations of each species currently is not fully documented. Given the wide distribution and highly-migratory nature of all these species, it is likely that useful global population assessment needs to be done through additive regional assessments.

While shark populations are poorly defined, shark-human interactions in the form of unprovoked attacks have been increasing in raw numbers for more than a century and in increasingly diverse places [12]. The increase in attacks—particularly in the United States, Australia, and South Africa—have been attributed to increases in human population and the subsequent increase in water-based recreational activities. In fact, although the claim of increasing shark attacks is often made in popular media, when adjusting for population growth the true risk may actually be declining in some places [14].

Given the confusion surrounding the interpretation of risk of shark interactions coupled with the importance that is placed on public safety, it is important to both understand the real risk of shark interactions and how that risk varies spatially and temporally (eg how regional risks have changed over decades). Simultaneous to improved public understanding of risk is the desire for an informed public in places where shark populations are threatened, such that unnecessary mortality is not added to depressed populations. Our objective was to quantify increase or decrease in annual probability of shark attacks at the country and regional level, and describe spatial variability in fatal outcomes and relative risk of different water-based activities to shark attack.

## Materials and methods

Our approach was to investigate two models for quantifying global trends in unprovoked shark attacks—a model based on the country in which an attack was reported and a model based on a region of a country in which an attack was reported. The regional-level analysis was based on a regionalization of habitats frequented by certain species of sharks. Although the country-level is useful for characterizing broad-scale trends, it often pools multiple habitats, multiple species, and even multiple oceans, which may mask species effects or smaller-scale patterns. For the country analysis, data were limited to the 14 countries with ≥10 shark attacks since 1960 (Table 1). For the regional analysis, country data were subset to include only those countries with ≥100 shark attacks (a value of 100 attacks was chosen to ensure adequate sample sizes within regions): the Commonwealth of Australia, Republic of South Africa and the United States of America. Regions were determined based on the dominant shark species involved in attacks, but also supported by the regional habitat (see Table 2 for regions and dominant species) because positive identifications of attacking sharks can often be difficult and uncertain.

**Table 1:** Fatal and non-fatal outcomes of shark attacks by country from 1960–2015. Non-fatal outcomes globally represented 85% of attacks.

Country	Fatal	Non-Fatal	*n*
USA	2%	98%	1215
Australia	15%	85%	315
Republic of South Africa	16%	84%	202
Brazil	22%	78%	88
New Zealand	12%	88%	42
Mascarene Islands	46%	54%	41
Mexico	48%	52%	33
Papua New Guinea	47%	53%	32
Bahama Islands	4%	96%	24
Fiji Islands	30%	70%	20
Egypt	29%	71%	14
Hong Kong	77%	23%	13
Ecuador	0%	100%	11
New Caledonia	60%	40%	10

**Table 2:** Descriptions of regions used (with corresponding country in parenthesis), the corresponding Map code in Fig 1, and the dominant attributed shark species for that region.

Region	Map Code	Dominant Species
Gulf of Mexico and Atlantic Coast (USA)	1	Blacktip, Spinner, Bull, Tiger sharks
West Coast (USA)	2	White shark
Hawaii (USA)	3	Tiger shark
Western Cape (South Africa)	4	White shark
Eastern Cape and KwaZulu-Natal (South Africa)	5	Bull shark
Northern Territory, Queensland (Australia)	6	Tiger shark
New South Wales, Victoria, South Australia, Western Australia, Tasmania (Australia)	7	White, Bull shark

### Shark attack data

We obtained all unprovoked shark attack data from the International Shark Attack File [12], the longest running and most inclusive data source on global shark attacks. We did not consider the outcome, with respect to severity of injury or fatality; such outcomes, which are likely time, location, and species-dependent, are not particularly relevant to our question. Location of attack was primarily of interest at the country and state/province scale. Specifics of location—such as water depth, temperature, and salinity—were not relevant to our question and also less certain than large-scale location data. All shark attacks were assumed to be by a single shark on a single individual (human). There have only been a few documented serial attacks attributable to so-called “rogue sharks.” Finally, we also examined victim activity to look for any indications that certain water-based activities are risker than others. Although the data presented a wide variety of reported activities, all activities were grouped based on common interactions with the water (e.g., kayaking, kitesurfing, and kiteboarding are grouped as surface activities while scuba diving, hookah diving, and hard-hat diving are all classified as prolonged underwater diving.) We acknowledge that there may be some reporting bias in the data and that our data represents the documented subset of all shark attacks. With that being said, the ISAF has numerous procedures in place to maximize detection of shark attacks around the globe and even historic attacks are constantly subject to revision and addition.

### Human population data

For our country-level analyses, we used annual World Bank population data (1960–2015) [15] and all areas with shark attacks corresponded to World Bank countries, with the exception of the Mascarene Islands (where data was not available) in which case the population for Mauritius was used. For our regional level analysis we drew population data from national sources. The three regions in the US were compiled from the US census data [16] for each state that has any coastline with a reported attack region. Population data for regions in Australia were compilations of populations of Australian states (Australian Bureau of Statistics, unpublished data). Province population data was not available for South Africa before 2002, so we modeled 2002–2015 projected population data (Statistics South Africa, unpublished data) using a linear model and extrapolated 1970–2001. All population data used in modeling was annual population estimates.

### The model

We employed a Bayesian hierarchical modeling framework to estimate global temporal trends in shark attacks. A zero-inflated Poisson distribution was assumed for the response variable (*y*), the number of shark attacks in location *i* in year *t*, where *y*_*i*,*t*_ ∼ *ZIP*(*μ*_*i*,*t*_, *π*_*i*_). A log-link was used for the expected value, where *log*(*μ*_*i*,*t*_) = *η*_*i*,*t*_ and a logit link for the probability of a false zero, where *logit*(*π*_*i*_) = λ_*i*_ (a diffuse normal prior was used for the logit-scaled probability of a false zero, ie λ_*i*_ ∼ *N*(0, 1000). The predictor for quantifying temporal trends was as follows:

$$\begin{array}{c} \eta_{i,t}={\text{offset}}_{i,t}+\phi_{i}+\nu_{i,t} \end{array}$$ (1)

Where offset_*i*,*t*_ is log_*e*_(annual population size) for location *i* in year *t*, *ϕ*_*i*_ is the site-specific intercept (a diffuse normal prior *ϕ*_*i*_ ∼ *N*(0, 1000) was used for each *ϕ*_*i*_), and *ν*_*i*,*t*_ is the site-specific trend. A Gaussian random walk prior of order 1 (RW(1)) was used for the temporal random effect. We used a 1D conditional autoregressive model for the temporal RW(1), where $\nu_{i,1:T}∼CAR\left(Q,\sigma_{i,\nu }^{2}\right)$ and $log\left(\sigma_{i,\nu }^{2}\right)∼N\left(\mu_{\sigma_{\nu }^{2}},\omega_{\nu }^{2}\right)$ A diffuse normal prior, *N*(0, 1000), was used for $\mu_{\sigma_{\nu }^{2}}$, the mean log site-specific variance and a diffuse uniform prior, *unif*(0, 10), was used for *ω*_*ν*_.

All models were fitted using WinBUGS [17], executed from within R [18]. Three parallel chains were run, each with 80,000 iterations, with different initial values to generate 240,000 samples from the posterior distributions. The first 50,000 samples of each chain were discarded and every third sample was retained, for a total of 30,000 samples (ie 10,000 per chain). We examined the scale reduction factor, a convergence statistic, for each parameter, trace plots, and plots of posterior distributions to assess convergence.

The change in shark attacks among years (Δ*Trend*) was calculated by subtracting the expected value for each site in year *t* from the expected value in year *t* + 1, across all MCMC samples. The probability of an annual increase in shark attacks from year *t* to year *t* + 1 was simply the average number of times, across all MCMC samples, that Δ*Trend* was greater than zero.

## Results

**Fig 1:**
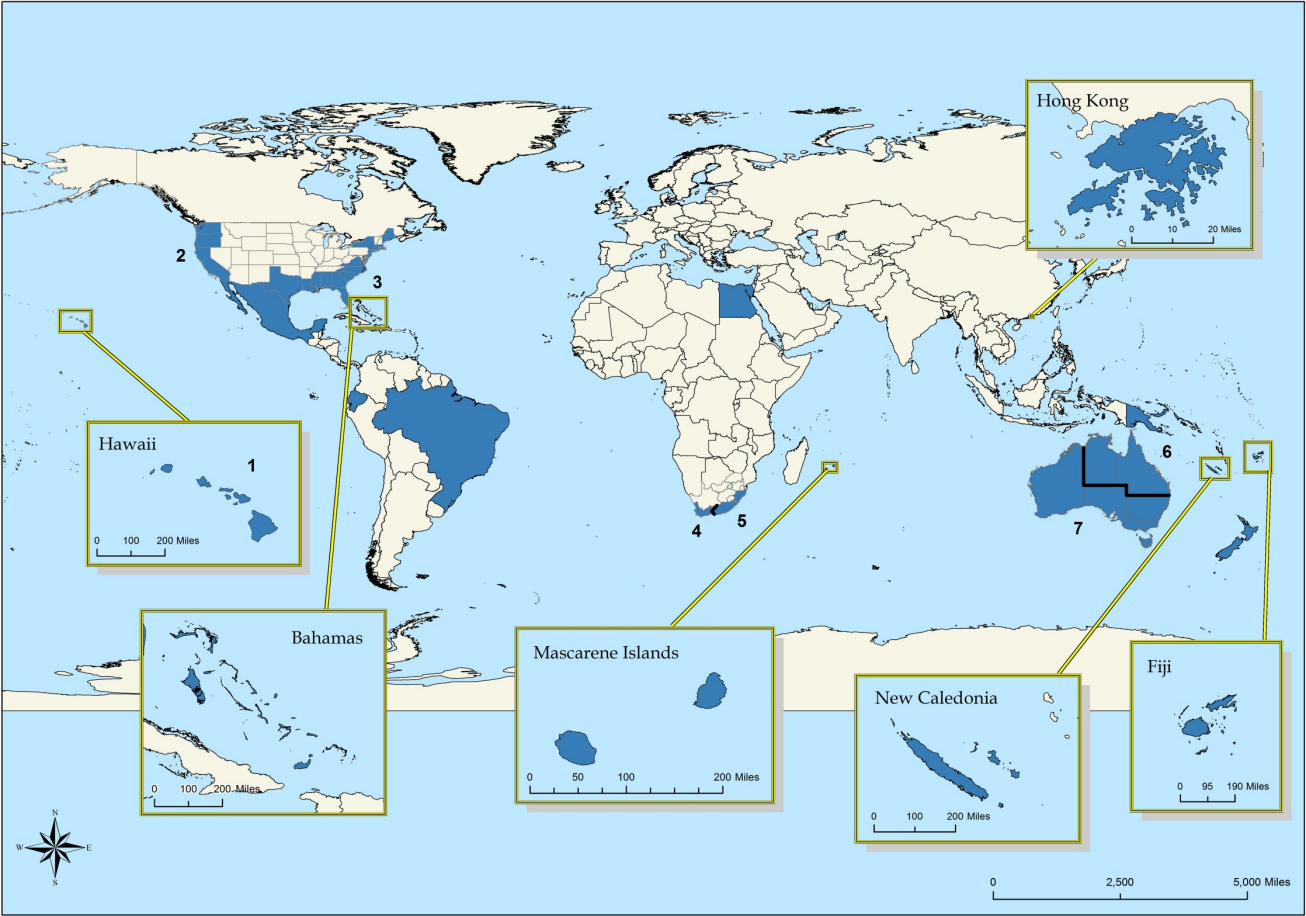
World map of countries (and regions) included in our analysis. Countries (*n* = 14) included are shown in blue, while regions are represented by numbers (*n* = 7) and are areas within the USA, the Republic of South Africa, or Australia.

A total of 14 countries were examined in the country-level analysis (Table 1; Fig 1), and seven regions (from a total of three countries) were examined in the region-level analysis (Table 2). Although not the focus of this contribution, the majority of all shark attack outcomes were non-fatal (85%), and typically less than about 25% of attacks were fatal in countries with >50 attacks since 1960.

**Fig 2:**
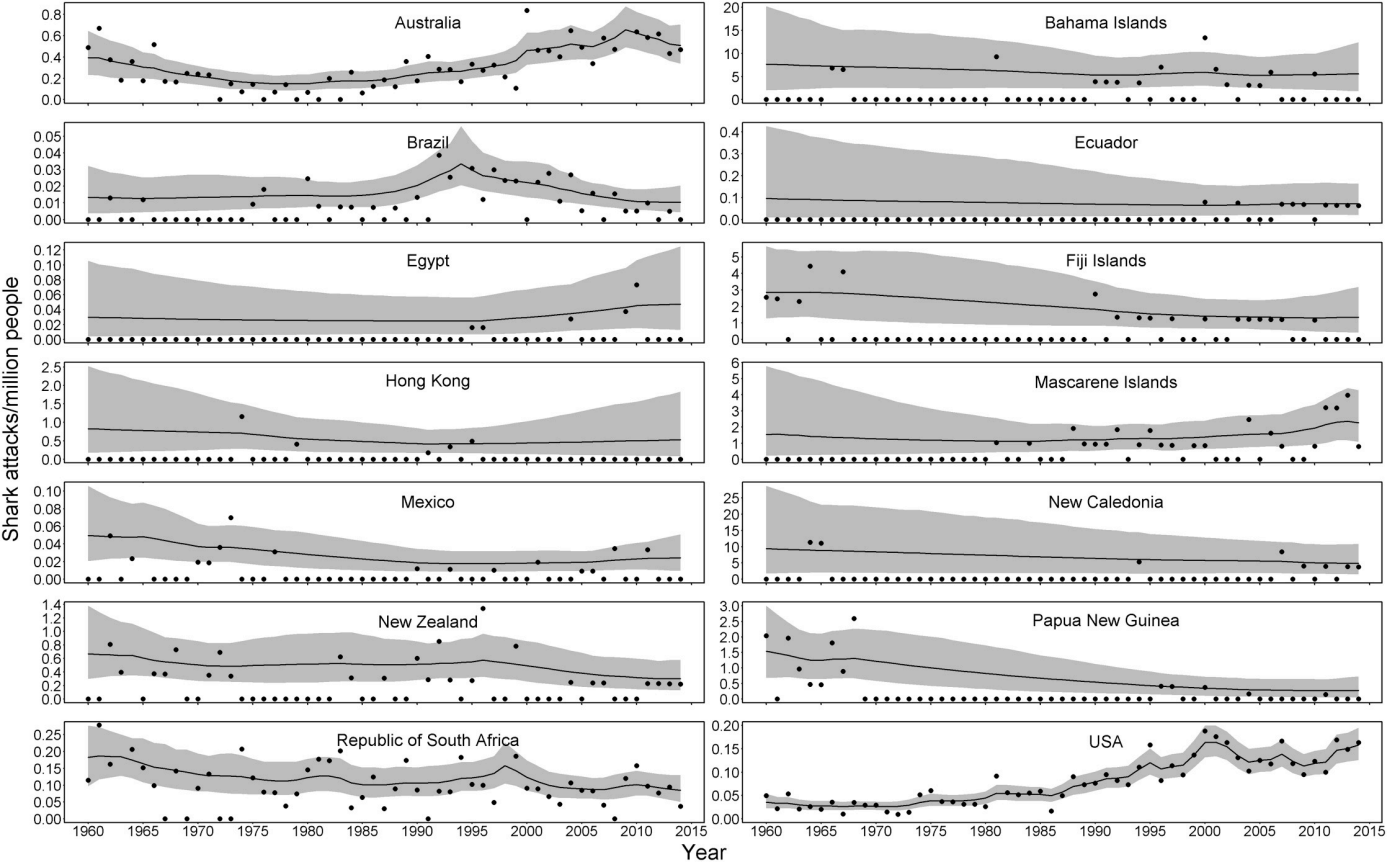
Shark attack rate (attacks per million people) from 1960–2015 for the 14 countries with the most shark attacks during this time. Black dots indicate annual rates, the black line represents the temporal trend, and the gray region indicates uncertainty (95% credible region) around the trend.

**Fig 3:**
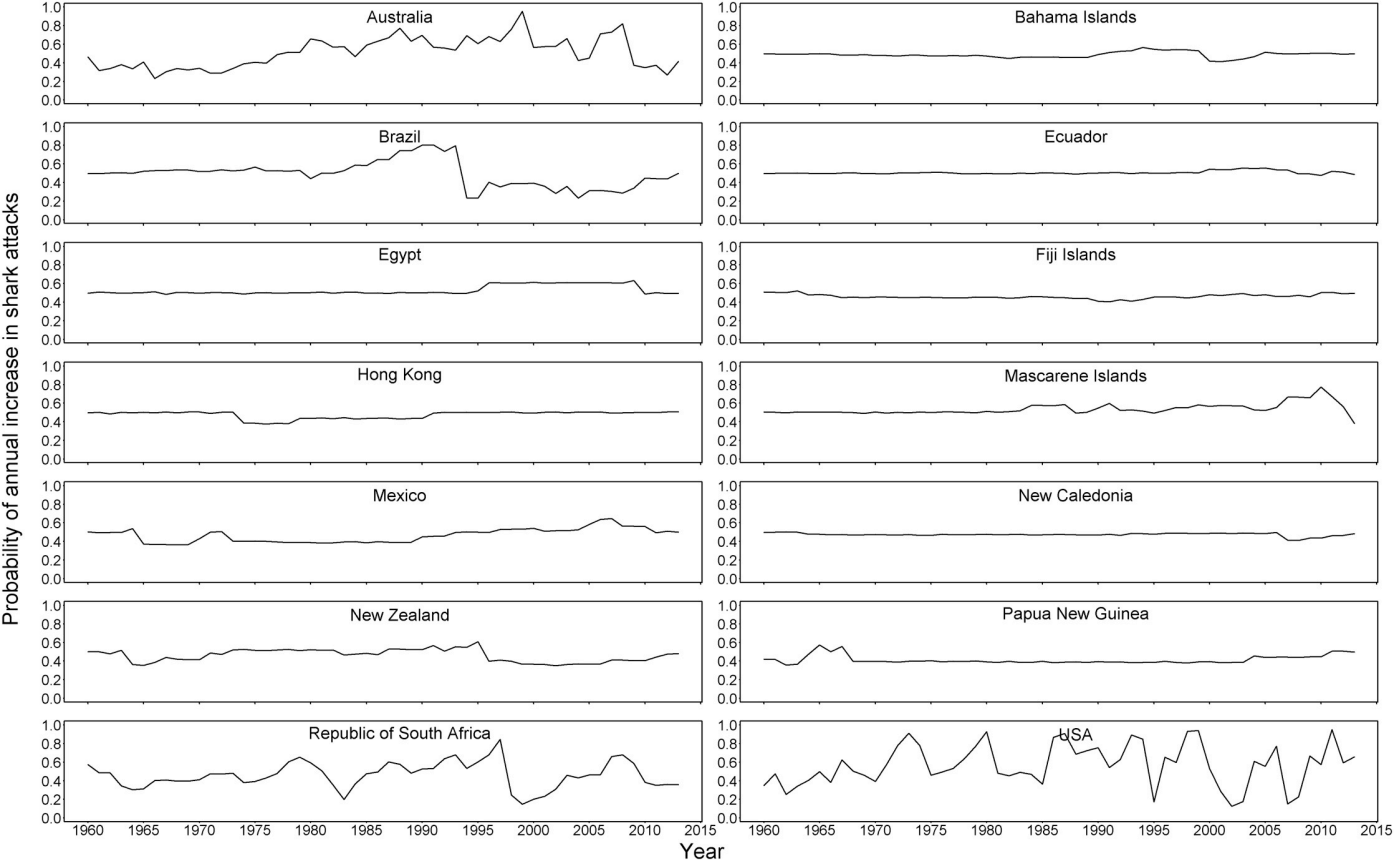
Annual probabilities of an increase in shark attack rate from 1960–2015 for the 14 countries with the most shark attacks during this time.

For the country-level analysis, the Bayesian hierarchical model detected considerable variability in temporal patterns. For example, countries like the Bahamas and New Caledonia had high attack rates (>5 shark attacks per million people), but the high rate is strongly influenced by a low population (and a lack of tourism data). Other countries like the USA and Australia had low attack rates (<1 shark attack per million people), but reported higher numbers of attacks. The majority of countries saw no perceptible trend or change in attack rates over decades (Figs 2 and 3), while some of the higher population countries like the USA and Australia exhibited increasing attack rates over time. Annual probability plots were much more sensitive to the number of attacks per year, but also much more variable in countries with higher numbers of attacks. Generally speaking, uncertainty increased as the mean number of annual shark attacks increased, a property of any Poisson-distributed response (Fig 3).

**Fig 4:**
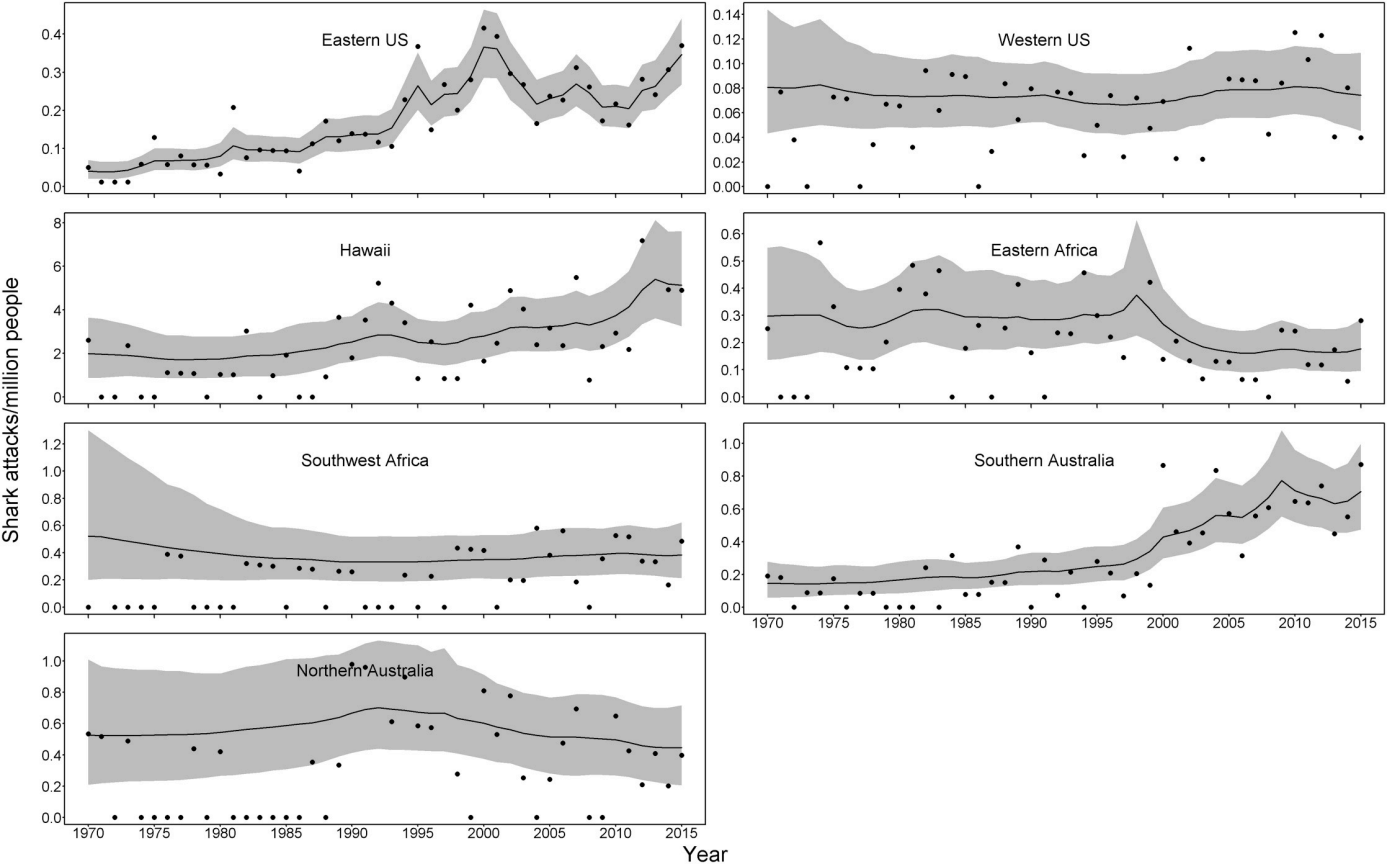
Shark attack rate (attacks per million people) from 1970–2015 for 7 regions in countries with the most attacks during this time. Black dots indicate annual rates, the black line represents the temporal trend, and the gray region indicates uncertainty (95% credible region) around the trend.

**Fig 5:**
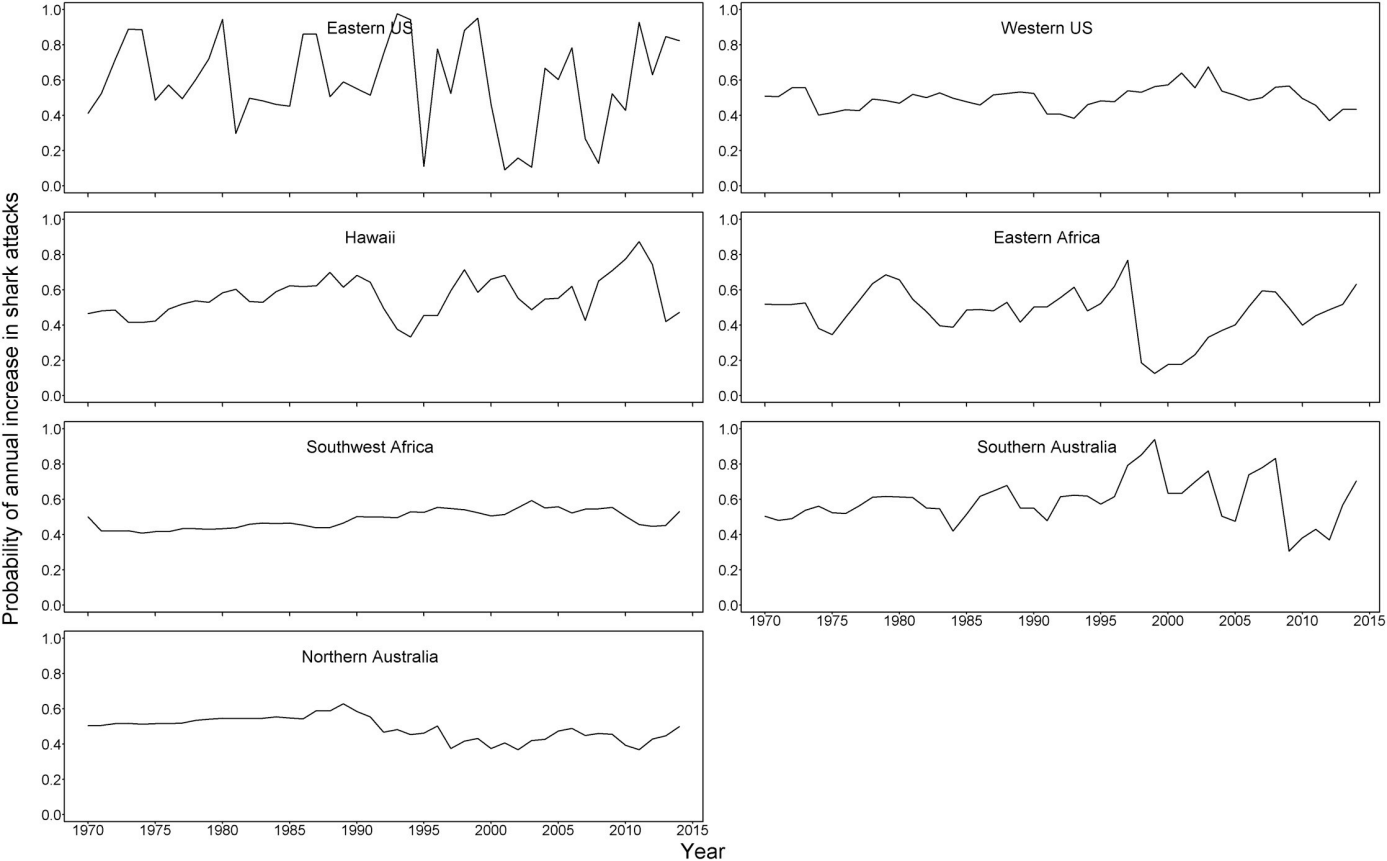
Annual probabilities of an increase in shark attacks rate from 1970–2015 for 7 regions in countries with the most attacks during this time.

The region-level analysis helped inform which regions may be influencing the country trends, and was also conducted to represent a more ecologically-relevant regionalization (because any given country may include different coasts and oceans dominated by different shark taxa). The region of southern Australia is driving most of the overall Australia trend, which makes sense as much of the human population and shark attacks occur in this region. The eastern U.S./Gulf of Mexico, Hawaii, and Southern Australia show noticeable increases in attack rates over time (Fig 4), but also with much of the increase occurring since the 1990s. Interestingly, these three regions, are dominated by a different shark species. Attacks in Hawaii are mainly from tiger sharks, Southern Australian attacks are mainly from white sharks, and the eastern US/Gulf of Mexico are a mix of bull sharks (which dominate fatalities) and blacktip and spinner sharks (in non-fatal attacks). Because regions were selected based on shark attack numbers we expected to see greater variation in the probability of annual increase in attacks, which is evident in Fig 5.

**Fig 6:**
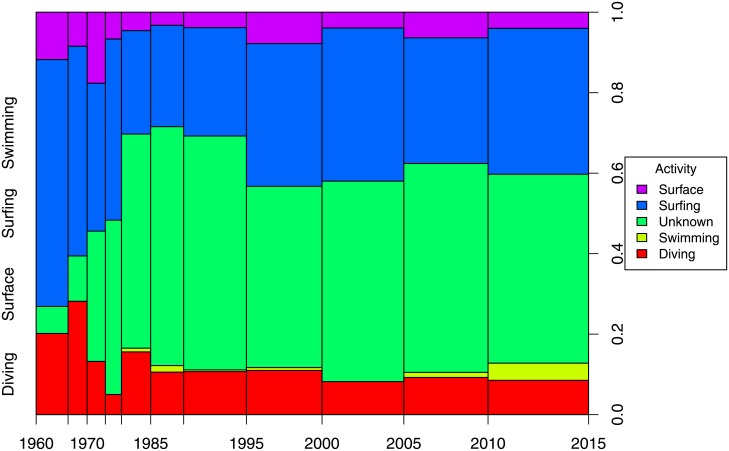
Proportion of shark attacks by victim activity over time from 1960–2015. (Diving includes Scuba diving, free diving and spearfishing; surface includes any surface or top water activities; surfing include boogie boarding and body boarding; swimming includes wading.) The widths of the 5-year periods are proportional to the sample size for the period, with thinner intervals indicating fewer samples (with 65 attacks for the period of 1975–1980 and 446 attacks for the period of 2010–2015, for reference).

Although not the focus of our analysis, we wanted to examine for any patterns of shark attack by victim activity over time. Considering four of the most common types of activities—diving, surface activity, surfing, and swimming— the proportions of shark attack by activity have remained relatively constant since 1980(Fig 6). Surfing and swimming dominated the activities undertaken immediately prior to a shark attack, with diving representing about 10% of the activities.

## Discussion

Predicting individual shark attacks is impossible, and was not our task. We were more interested in long-term trends and investigating regional trends based on dominant species attributed in attacks. There is value in understanding both the regional risks associated with water-based activities, and perhaps more value in being able to interpret reports of high or low attacks in a given year. As expected, regions with very few attacks (but not necessarily low attack rates) tend to not change much annually. In regions with greater numbers of attacks, the probability of one year being different from the year before it can be minor, but also very large; for example, the eastern US/Gulf of Mexico has seen probabilities of annual increases in shark attacks fluctuate from nearly 0 to 1 between just one year, and changes >0.5 are common. Generally speaking, greater raw numbers of attacks means more data and therefore better estimation of uncertainty—you can’t have a very high or low probability of annual change if you are very uncertain about the attack rate because there are few attacks. For example, Australia and the USA both have increasing trends and less uncertainty than other countries, which means that their probabilities of annual increase in attacks is better estimated. The upshot is that areas with large numbers of attacks tend to be the most variable year-to-year.

Although our work is not the first to report on shark attacks, ours is the first to do so globally over half of a century. Other studies into shark attacks have reported similar results—namely, that the USA, Australia, and the Republic of South Africa account for a large proportion of shark attack locations, and that white sharks, bull sharks, and tiger sharks are the primary species involved in attacks [19, 20]. Our work differs from other’s [19] in that a different shark attack database was used and only linear models were applied to detect trends. Although we do not question the trends that were reported, our models provided greater insight by accounting for the autoregressive nature of the data and the quantifying of the probability of a trend. McPhee (2014) also reported that while increases in human population may account for some increase in shark attacks, it does not account for it all. It is likely that shark populations, coastal development, and environmental conditions (among other factors), influence shark attack rates [20]. We did not include environmental factors or shark population data in our model—largely because this data is incomplete at the global scale—but an annual investigation into shark-environment correlations and subsequent attacks may be useful.

We split regions based on dominant shark species in the attack records (which matched with our expectation based on habitat). However, shark identification is often unreliable during an attack, and some regions host more potential attacking species than others. For example, attacks in Hawaii are almost exclusively tiger sharks, while attacks in the eastern US/Gulf of Mexico nominally is dominated by bull shark attacks, but less easily identified and uncredited blacktip and spinner sharks probably are more often involved along with other difficult to identify requiem sharks of the family Carcharhinidae. In all regions there are attacks that do not report a species, and even when reported, many identifications are not certain.

### Effect of tourism

We were forced to use human population as a surrogate human unit effort with full knowledge that not all humans enter the sea or do so with the same frequency or duration; for example, wading may an activity that averages minutes, while surfing averages hours. Data documenting human-hours spent in the sea simply does not exist. Similarly, although some explicit tourism estimates may be available for some locations in some years, the global and long-term nature of our data required that we use a common proxy for tourism in all sites. Tourism data would represent an improvement to our model; however, 1) tourism data does not exist over the spatio-temporal scope of our study, and 2) many of these countries and regions have high and variable amounts of tourism, so it is difficult to draw any strong conclusions without tourism data. Additionally, while more tourism may result in more attacks, the relationship may not be linear. For instance, there may be a threshold effect of tourism—smaller increases in tourism may not change any attack rates, but once tourism exceeds a certain level, attack rates may increase. In other words, you need a certain number of bodies in the water just to get to 1 or 2 shark attacks, but when you have consistently high numbers, other factors drive the relationship. A threshold may also explain trends observed in the eastern US/Gulf of Mexico, Hawaii, and Southern Australia. These three areas report stronger increases in attack rates beginning in the mid-1990s (and Hawaii more recently). Because there is currently no clear explanation (although some unquantified explanations exist, such as an increase in surfing, which may be considered a provocative behavior) as to why attack rates increased in these areas, it could be that tourism numbers cross a threshold in each of these areas, whereby the attack rate took on a different relationship to humans in the water. Additional evidence of tourism may come from the presence of wobbegong (Family Orectolobidae) bites in Australia—wobbegong typically only bite when stepped on, and more humans in the water likely drives the recent increase in these attacks. (It should be noted that other hypotheses for these changes could also be entertained, such as population trends of coastal shark populations in these areas). Generally speaking, the effects of tourism are likely complex, including the potential for effects of dynamics of internal human migration (with state or province), variation in user hours (more surfers mean a lot more user hours, while more divers mean more restricted hours based on tanks time), and water temperature.

### Importance of smaller scales

Large spatial scale description of shark attacks has value in understanding trends; however, conditions at smaller scales (county or beach-scale) may better inform user decisions regarding water interactions. For example, California (U.S.A. west coast) is unique in that while we might expect to see a signal because high population, the attack rate is likely depressed due to several factors. Namely, 1) any effect of surfing probably began in the 1950s (before the time series), 2) the water is generally cold which keeps large numbers of users out of the water, and 3) with white sharks being the dominant species, they tend to be less numerous than other attacking shark species. Similarly, increases in tourism may get to a point where more preventative measures are taken which can level off or even decline attacks. (e.g., Brazil, South Africa), ultimately masking the effect of increasing tourism. Countries also see popularity trends in water-based activities, which may result in changes in either attack rates or fatal outcomes. For example, the Mascarene Islands has seen recent increases in surfing, while diving has increased in Egypt. Humans are also now able to access remote locations with greater ease than in the past, and such remote locations may also pose an inherently different risk than a crowded beach, for instance.

## Conclusions

Overall, trends in global shark attack rates vary substantially, are a function of many social-ecological interacting factors, and remain quite low. Despite the cultural perception of shark attack risk, the risk at larger scales is not very high, and where it is increasing the rates are low and preventative measures are more likely to take place. However, in highly populated regions—like the Eastern USA and Southern Australia—shark attack rates have doubled in the last 20 years, and while the rates remain relatively low, they should continue to be monitored. In locations with high raw number of attacks, any given year (or season) with an elevated or decreased number of attacks might not be that unusual, given the nature of any Poisson-distributed responses in which the variance increases directly to the mean. Ultimately, all shark attacks are local—risk is best assessed at the spatial and temporal scale of the beach or water body intended to be used and known risk factors at those scales should be considered over larger-scale patterns. Our work has shown that in some global locations, shark attacks are increasing over long time periods and are highly variable, and we recommend additional research at multiple scales to better articulate possible scale-dependent predictors of shark attacks.

## Supporting information

S1 Data Data used in this study. Variables include *water area (ocean)*, *continent*, *country*, *state*, *county*, and *year*.
(CSV)

## References

[1] Karl SA, Castro ALF, Lopez JA, Charvet P, Burgess GH. Phylogeography and conservation of the bull shark (*Carcharhinus leucas*) inferred from mitochondrial and microsatellite DNA. Conservation Genetics. 2010;12(2):371–382. 10.1007/s10592-010-0145-1

[2] Bethea DM, Ajemian MJ, Carlson JK, Hoffmayer ER, Imhoff JL, Grubbs RD, et al Distribution and community structure of coastal sharks in the northeastern Gulf of Mexico. Environmental Biology of Fishes. 2014;98(5):1233–1254. 10.1007/s10641-014-0355-3

[3] Heupel MR, Knip DM, Simpfendorfer CA, Dulvy NK. Sizing up the ecological role of sharks as predators. Marine Ecology Progress Series. 2014;495:291–298. 10.3354/meps10597

[4] Roff G, Doropoulos C, Rogers A, Bozec YM, Krueck NC, Aurellado E, et al The Ecological Role of Sharks on Coral Reefs. Trends in Ecology and Evolution. 2016;31(5):395–407. 10.1016/j.tree.2016.02.014 26975420

[5] Winemiller KO, Rose KA. Patterns of life-history diversification in North American fishes: implications for population regulation. Canadian Journal of Fisheries and Aquatic Sciences. 1992;49(10):2196–2218. 10.1139/f92-242

[6] Clarke SC, McAllister MK, Milner-Gulland EJ, Kirkwood G, Michielsens CG, Agnew DJ, et al Global estimates of shark catches using trade records from commercial markets. Ecology letters. 2006;9(10):1115–1126. 10.1111/j.1461-0248.2006.00968.x 16972875

[7] Dulvy NK, Baum JK, Clarke S, Compagno LJ, Cortés E, Domingo A, et al You can swim but you can’t hide: the global status and conservation of oceanic pelagic sharks and rays. Aquatic Conservation: Marine and Freshwater Ecosystems. 2008;18(5):459–482. 10.1002/aqc.975

[8] Baum JK, Myers RA, Kehler DG, Worm B, Harley SJ, Doherty PA. Collapse and conservation of shark populations in the Northwest Atlantic. Science. 2003;299(5605):389–392. 10.1126/science.1079777 12532016

[9] Baum JK, Myers RA. Shifting baselines and the decline of pelagic sharks in the Gulf of Mexico. Ecology Letters. 2004;7(2):135–145. 10.1111/j.1461-0248.2003.00564.x

[10] Burgess GH, Beerkircher LR, Cailliet GM, Carlson JK, Cortés E, Goldman KJ, et al Is the collapse of shark populations in the Northwest Atlantic Ocean and Gulf of Mexico real? Fisheries. 2005;30(10):19–26. 10.1577/1548-8446(2005)30[19:ITCOSP]2.0.CO;2

[11] Simpfendorfer CA, Dulvy NK. Bright spots of sustainable shark fishing. Current Biology. 2017;27(3):R97–R98. 10.1016/j.cub.2016.12.017 28171764

[12] ISAF. International Shark Attack File; 2016.

[13] Muter Bret A and Gore Meredith L and Gledhill Katie S and Lamont Christopher and Huveneers Charlie Australian and US news media portrayal of sharks and their conservation Conservation Biology. 2013;27:187–196 10.1111/j.1523-1739.2012.01952.x 23110588

[14] Ferretti F, Jorgensen S, Chapple TK, De Leo G, Micheli F. Reconciling predator conservation with public safety. Frontiers in Ecology and the Environment. 2015;13(8):412–417. 10.1890/150109

[15] World Bank. The World Bank: Population Data; 2016.

[16] United States Census Bureau. United States Census Bureau: Population; 2016.

[17] Lunn DJ, Thomas A, Best N, Spiegelhalter D. WinBUGS—a Bayesian modelling framework: concepts, structure, and extensibility. Statistics and Computing. 2000;10(4):325–337. 10.1023/A:1008929526011

[18] Team R. R: A language and environment for statistical computing. R Foundation for Statistical Computing, Vienna, Austria. 2014; 2016.

[19] McPhee D. Unprovoked shark bites: Are they becoming more prevalent? Coastal Management. 2014;42:478–492 10.1080/08920753.2014.942046

[20] Chapman BK, McPhee D. Global shark attack hotspots: Identifying underlying factors behind increased unprovoked shark bite incidence Ocean & Coastal Management. 2016;133:72–84 10.1016/j.ocecoaman.2016.09.010

